# Suffering, hope, and entrapment: Resilience and cultural values in Afghanistan

**DOI:** 10.1016/j.socscimed.2010.03.023

**Published:** 2010-07

**Authors:** Mark Eggerman, Catherine Panter-Brick

**Affiliations:** Durham University, Durham DH1 3HN, United Kingdom

**Keywords:** Afghanistan, Mental health, Resilience, Social justice, Psychosocial wellbeing, Conflict, Suffering, Violence

## Abstract

A critical health-related issue in war-affected areas is how people make sense of adversity and why they show resilience in a high-risk environment. In Afghanistan, the burden of poor mental health arises in contexts of pervasive poverty, social inequality, and persistent violence. In 2006, we conducted face-to-face interviews with 1011 children (age 11–16) and 1011 adult caregivers, randomly selected in a school-based survey in three northern and central areas. Participants narrated their experiences as part of a systematic health survey, including an open-ended questionnaire on major life stressors and solutions to mitigate them. Responses were analysed using an inductive thematic approach and categorised for quantitative presentation, producing a conceptual model. For adults, the primary concern is repairing their “broken economy,” the root of all miseries in social, educational, governance, and health domains. For students, frustrations focus on learning environments as well as poverty, as education is perceived as the gateway to upward social and economic mobility. Hope arises from a sense of moral and social order embodied in the expression of key cultural values: faith, family unity, service, effort, morals, and honour. These values form the bedrock of resilience, drive social aspirations, and underpin self-respect and dignity. However, economic impediments, social expectations, and cultural dictates also combine to create entrapment, as the ability to realise personal and social aspirations is frustrated by structural inequalities injurious to health and wellbeing. This study contributes to a small but growing body of work on resilience in public health and conflict settings. It demonstrates that culture functions both as an anchor for resilience and an anvil of pain, and highlights the relevance of ethnographic work in identifying what matters most in formulating social and public health policies to promote a hopeful future.

## Introduction

Life feeds on hope (mother, age 28, Kabul)

Hope… is not the conviction that something will turn out well, but the certainty that something makes sense, regardless of how it turns out (Václac Havel, 1990: 181)

In war-affected areas, *resilience* is a critical issue for research, humanitarian policy, and public health. It is the next frontier of knowledge in the field of violence and health, an intuitively useful construct to explain why, despite significant exposure to war, individuals and families achieve emotional adjustment and social functioning, namely “good adaptation in a context of risk” (Mastern, 2006: 4), while communities and state structures withstand recurrent conflict (Betancourt & Khan, 2008; Panter-Brick, 2010; Pedersen, 2002). Despite a hefty literature on resilience in Western journals, calls have been made for rigorous and ethnographically-grounded approaches to resilience research in public health (Wexler, DiFluvio, & Burke, 2009), mental health (Almedom & Glandon, 2007), and youth-focused studies of war experiences (Barber, 2009). The need to document how people ‘make sense of’ and ‘cope with’ life events is particularly acute in conflict zones, where much of the mental health literature has focused on risk rather than resilience, and overlooked how specific cultural and ecological contexts frame individual and collective experiences of political violence (Barber, 2009; Boyden & de Berry, 2004). For example, a contrast in ‘meaning-making’ underpins psychological responses of Bosnian and Palestinian youth, the former expressing shock at the deadliness of war, the latter expressing strong ideological commitment (Barber, 2008; Punamaki, 1996). Similarly, the links between mental health and protracted war experiences are mediated by mundane aspects of social ecology, expressed in a sense of community belonging and/or marginalisation and structural impediments to emotional and/or social functioning in everyday life (Miller & Rasmussen, 2010; Panter-Brick, 2010). Clearly, a more fine-grained portrayal of how individual psychology, cultural affiliation, and societal conditions mediate the impacts of violence on wellbeing is required (Barber, 2009; Seginer, 2008).

In Afghanistan, political and military conflicts have led to massive disruptions of livelihoods, education, and networks of social support. Afghan families endure pervasive poverty, economic instability, and persistent violence (Rubin, 2006; UNDP, 2004). Since the fall of the Taliban regime in 2001, and in the context of an ongoing war, large-scale reconstruction programmes have raised expectations for socio-economic advancement, accentuated inequalities, and led to widespread frustrations with persistent social injustice (Donini, 2007). How do Afghans experience such adversity, construct hopes for the future, and make sense of everyday suffering?

Previous epidemiological surveys have documented a high prevalence of mental health problems in adults, associated with exposure to traumatic events (Cardozo et al., 2004; Scholte et al., 2004). More recent research, integrating perspectives from psychiatry and anthropology, has shown that adult and child mental health correlates not just with past experiences of conflict, but with present-day stressors such as ongoing domestic violence and inequalities in access to basic services (Miller, Omidian, Rasmussen, Yaqubi, & Daudzi, 2008; Panter-Brick, Eggerman, Mojadidi, & McDade, 2008). In previous publications, we unpacked the nature of ‘trauma’ in Afghanistan – present in multiple forms, as militarized, community-based, and family-level violence – and revealed consistent associations between child-caregiver wellbeing (Panter-Brick, Eggerman, Gonzalez, & Safdar, 2009). We also examined the embodiment of psychosocial stress and the salience of family-level stressors (Panter-Brick et al., 2008). In this paper, we examine how sources of distress and resilience are articulated in Afghan families, in light of a theoretical understanding of suffering and hope derived from the social sciences.

The *anthropology of suffering* is a body of work that gives voice to the physical and emotional pain of people battling with chronic poverty, social marginalisation, and routinized violence. It focuses on disruption and regulation: how ordinary social life ‘hurts’ and how this hurt becomes part of the social experience (Davis, 1992: 155). Notions of *social suffering* are increasingly developed within medical anthropology, social history, cross-cultural psychology, and medical sociology (Kleinman, Das, & Lock, 1997; Scheper-Hughes & Bourgois, 2003; Stein, Seedat, Iversen, & Wessely, 2007). Analyses focus on narratives of pain and loss, cultural representations and moral understandings of the world, and the embodiment of structural violence, defined as insidious social, economic, legal and political barriers that put individuals in harm’s way (Farmer, 2004). In Palestine, for instance, socio-political stressors are significant determinants of personal wellbeing, and “poverty and powerlessness are just as salient as war events in shaping experiences of trauma” (Almedom & Summerfield, 2004: 385). Notions of social suffering thus link health problems with social problems, and individual with collective experiences: in conflict settings, they place social, cultural, political, and economic matters at the forefront of public health concerns (Pedersen, 2002: 186–7).

The *anthropology of hope* also links wellbeing with social structures, but receives comparatively less attention. Ideologies of hope have significance for individual and collective resilience, social identity, and social dynamics across successive generations (Carbonella, 2003; Loizos, 2008; Miyazaki, 2004). Hage (2003) forcefully argued that in contexts of marked inequality, society is a mechanism not only for the distribution of social opportunities, but also for the distribution of *social hope*: access to resources reduces or encourages dreams of social mobility. Such hope is about “one’s sense of the possibilities that life can offer… Its enemy is a sense of entrapment, of having nowhere to go, not a sense of poverty” (p. 20). In Havel’s critical definition of the term, hope is not the illusion of a favourable outcome in the future, but “the certainty that something makes sense” (Havel, 1990: 181), a coherent narrative that explains personal and collective experiences. Arguably, this is what matters most in efforts to promote mental health, psychosocial support, and psychosocial wellbeing in war zones (Almedom & Glandon, 2007). One evidence-based review singled out ‘hope’ as one of five recommended elements for interventions addressing trauma resulting from mass violence, along with a sense of safety, calm, connectedness, and self/community-efficacy (Hobfoll et al., 2007). Thus hope is clearly identified as an important component of resilience. However, there is little critical examination of how hope is articulated in settings of collective and structural violence.

Using the framework of a school-based mental health survey (Panter-Brick et al., 2009), this paper presents a thematic analysis of narratives of adversity, suffering, and resilience in Afghanistan. We analyse how constructs of suffering and hope frame cultural understandings of life experiences, in response to everyday stressors and professed solutions. We also critically examine why and how culture matters in shaping experiences of distress and resilience, in generating both a sense of coherence and a sense of entrapment. Two original aspects of this work were to draw a systematic random sample of participants for in-depth interviews, thus transcending many limitations of purely qualitative work, and to provide a cross-generational perspective, through statements from both children and adults.

## Study design

In 2006, we conducted a school-based health survey of 11–16 year old students and their caregivers in the central and northern municipalities of Kabul, Bamyan, and Mazar-e-Sharif; for security reasons work was not possible in southern Afghanistan. Schools were the best point of contact for drawing a community-level sample: they gave access to both female/male participants and a safe context for research activities, with time to build rapport and space for interview privacy. School attendance has grown exponentially in central/northern areas since the ‘Back to School’ campaign initiated in 2002: nationally, enrolment was 64% for 7–14 year-olds (48% girls, 77% boys) in 2004–2005 (Bakhshi & Trani, 2006), and institutions struggle to cope with the influx of students (Hunte, 2006). We could not overcome barriers to systematically sample families who did not, or would not, send their children to school, a limitation of this study. We aimed to contact children old enough to narrate their life experiences.

We randomly selected 10% of schools in each area, with probability sampling proportional to size and equivalence of boy/girl schools, including both city-centre *lycées* and Islamic *madrassa*. We then used class enrolment lists to randomly select 40 students per school, totalling 5% of attendees in age-relevant grades. Students contacted their principal caregiver to meet for interview over a 10 day-period/school. In total, we selected 1260 students and met with 1021 (81%) caregivers. Excluding 1 participation refusal and 9 cases with missing information, the sample comprised 1011 student/caregiver dyads in 35 schools (Panter-Brick et al., 2009).

Our research team moved sequentially from school to school, to ensure data quality and comparability. The project manager had international research experience, long-term residence in Afghanistan, and fluency in English and Dari. Six experienced Afghan interviewers (three male, three female) and one professional translator were recruited in Kabul. Both authors participated in staff training, survey piloting, and initial phases of data collection. Research was approved by the Durham University Ethics Committee and the Ministry of Education in Afghanistan. Participant informed consent was secured in writing from school directors and verbally from respondents.

Following drawing sessions and health checks to build rapport with participants, students and caregivers were interviewed – separately – in quiet locations on school grounds. Our survey covered physical health, lifetime exposure to traumatic events, psychometric assessment of mental health, and social functioning. This paper presents data from a semi-structured questionnaire designed to appraise psychosocial wellbeing and life adversity more broadly, which we called ‘Problems and Solutions.’ Simple questions (Table 1) prompted respondents to identify their main problems, no matter how big or small, their most distressing problem in daily life, and what might be done to improve their situation. Ample opportunity was given to explain responses in depth; statements were written down verbatim in Dari/Pashto.

## Analyses of textual data

Interviews were translated and checked by field staff in Afghanistan. The first author, able to read Dari and Pashto script, reviewed translations and transcribed to electronic format, case by case, all materials regarding the 1011 child/caregiver dyads. Respondent data were reviewed in English by both authors and analysed using inductive thematic analysis (Bernard, 2006; Pope, Ziebland, & Mays, 2000). We searched the text for themes, marking up verbatim statements in vernacular and English language, and iteratively examined text to extract thematic patterns (Table 1). We then contextualised statements in light of respondent health and trauma histories.

After content analysis, we categorised ‘problems’ and ‘solutions’ for quantitative presentation across generation and gender, as well as geographical area. Our categories captured the essence of verbatim expressions: problems related to the economy, housing, health, education, social relationships, and governance, while solutions, where envisaged, related to action by self, relatives, and government. An independent researcher, blind to study aims, checked coding against English translations. Our purpose was to illustrate top-of-mind responses, rather than statistically evaluate frequency data: we had recorded open-ended statements, not responses to fixed alternatives.

Together, thematic analyses and systematic coding provided the basis for a conceptual model of suffering, resilience, and entrapment.

## Overview of problems

We obtained a gender-balanced sample: 503 male and 508 female students [age 13.5 (SD 1.6) years] and 503 male and 508 female caregivers [age 36.5 (SD 12.3) years] including 628 parents and 383 other close relatives with direct responsibility for childcare. Only 30% of students had ever left Afghanistan, although 83% had been forcibly displaced and 9% had lived in refugee camps.

**Fig. 1:**
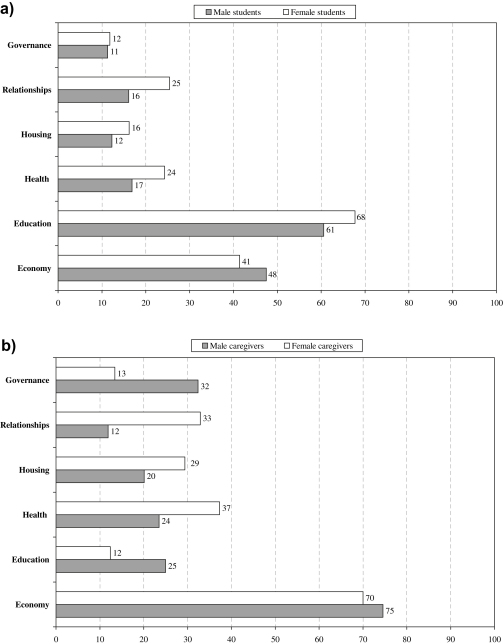
**a & b:** Types of problems reported by 1011 students and 1011 caregivers (% of responses) (a) students, by gender (b) caregivers, by gender.

Most respondents cited multiple problems; just 5% of adults and 10% of children cited no major worries in life (Table 1: Q1). The most commonly-cited stressors by caregivers were economic, while for students, educational stressors came ahead of the economy; this rank order pertained to both male and female respondents (Fig. 1). Adult men were more likely than women to raise governance and educational issues, while adult women were more likely to cite housing, relationships, and health. The rank order was similar for analyses of the *most* distressing problem (Table 1: Q2) and across areas (data not shown).

## Everyday stressors and social suffering

We’re not a very complex people. All we have to do is find a job, get a salary, and support the family. Caregiver (father, age 40).

### Poverty and the “broken economy”

Thematic analyses showed that different types of stressors were closely intertwined, but the fundamental problem was economic. As one father put it, “lack of work is the root of all a man’s miseries.” Most problems were explained by the Dari expression *iqtisad kharab* [lit: broken economy] and other catch-phrases that underlined the importance of money: *kam puli, kam nani* [lit: no money, no bread] and *pul pulra paida mekunad* [lit: money finds money, meaning “it takes money to make money”]. Men and women worried about financing a family, the lack of work, unstable markets, rising rents, large debts, and a frustrating inability to get beyond a state of simply “living from one day to the next” [*shab wa rooz megzarad* – lit. days and nights pass by].

The centrality of steady employment and income to the family’s sense of stability and wellbeing is evident in the following statement:

I don’t own my own home. I’ve got two sons, they’re both young men now, and I don’t have money to marry them off. My husband is a taxi driver, he only earns 150–200 Afs [$3–4 US] a day. There are fourteen people in our family, he can’t make enough to provide for us. We can barely make the rent. One of my sons died a year and a half ago, he had a tumour in his ear and he had typhus, he was only in fifth grade. If we’d had money for medical treatment he’d still be alive. Caregiver (mother, age 40).

Children echoed these frustrations – in our sample, two in ten students worked in addition to attending school. The strain born by these young workers is evident in the following:

We don’t have money. One day I have work, the next day I don’t. Sometimes I can’t sell fruit in the market and I don’t have any money… now all I’ve got is 500 Afghanis [$10 US]. Sometimes the tire on my cart goes flat, and I have to pay to get it fixed… There is no way you can earn enough to eat by selling fruit off a cart. Student (boy, age 16).

### Housing and “homelessness”

A key sign of a “broken economy” was not owning a home. The “homeless” [*be-khanagi*] included families who rented accommodation, shared a house with home-owning relatives, or lived as tenant farmers. Not owning one’s home was equated with a loss of social position and feelings of insecurity: one woman went as far as to state that she was willing to go hungry, as long as she owned the house that she lived in, while a 13-year-old girl said that owning a home was “more important than exams… if you don’t have a place of your own to live in, what good is school?” Home ownership was fundamental to a sense of stability, particularly in Kabul, where tenants were vulnerable to unpredictable rent rises and unscrupulous landlords:

We were living in the centre of town in a rented house. The landlord was living there with us and one day he just kicked us out because he said we were washing too many clothes. He didn’t even give us time to move our belongings out. It upset me a lot. If we’d had our own house, nobody would be able to do that to us. Student (boy, age 16).

### Social relationships: domestic violence and marginalisation

Poverty and living in overcrowded housing led to a deterioration of social relationships. Economically-frustrated husbands were described by their wives and children as being “ill-natured” [*bad khalqi*] – a socially neutral phrase indicating difficult or abusive domestic relations. Men’s outbursts of anger were put down to an inability to secure work and fulfil their responsibilities as household head. Violent behaviour at home was also labelled a “mental problem” [*takleef asabi*]. A 16-year-old girl linked money and job issues to poor health and abuse as follows: “my father’s salary is not enough for us, he has *takleef asabi* and he beats us…. if he finds a decent job then maybe he will calm down.” Women also reported being violent due to frustration with their circumstances:

My husband’s a driver, but he doesn’t own the car he drives, so he has to give a large part of what he earns to the owner. We have to share a house with four other families, we live in the separate rooms of the house and it’s difficult. My mind gets weaker and weaker, and I get upset and beat the kids. Yesterday I beat my daughter, then I felt bad about it and slapped myself on the face. Caregiver (aunt, age 28).

Social functioning was particularly difficult for widows and orphans. For widows, restrictions on female mobility in the absence of a male ‘chaperon’ [*mahram*] meant that their eldest child had to generate an income. They cited their lack of a male ‘guardian’ [*sarparast*] as their most distressing problem, even when residing within an extended household, due to their lack of status and influence over decision-making. Orphans, without a father/grandfather to protect their interests, were also in a weak social position, even when cared for by a paternal uncle. They might be compelled to stop school to work for the household, or be promised in marriage to less desirable partners relative to their male cousins (direct competitors in cross-cousin marriage arrangements).

### Ill-health

Poverty and its social ramifications placed a significant burden on the physical and emotional wellbeing of children and adults. Complaints about the cost and adequacy of local healthcare provision were frequent: as one 42-year-old mother bluntly stated, “you can’t [even] find out what your blood pressure is unless you have money.” Health, finance, and social problems exacerbated one another, especially if a breadwinner was lost to illness or disability. Children expressed strong anxieties about the economic and social destabilisation such a loss would bring.

Idioms of psychological distress were rooted in the body, and clearly differentiated between anger, stress, melancholy, and anxiety. *Takleef asabi* [lit. disorder of the nerves] indicated irritability and anger. The terms *fishar payin/fishar bala* [lit: low/high pressure] described lethargy and agitation as well as blood pressure. *Jighar khun* [lit. liver-blood – sorrow, regret, depression] referred to a state of acute dysphoria, often due to losing relatives as a result of war, while *tashwish* denoted everyday worry. Expressions such as *delam naram hast* [lit: my heart is noisy] or *delam az-zindagi sard shoda hast* [lit: my heart has become cold from life] were common, tied to feelings of embarrassment [*sharmandeh*], loss of honour [*‘izzat*] and frustration [*na’amedy*]. Chronic headaches, fatigue, fainting, and generalised body pain were linked to socio-economic stressors:

I get headaches because I have *fishar payin*. Sometimes my hands tremble or become numb, as if they have fallen asleep. I’m not happy because I’ve got money problems… and what can I do if my hands are empty? I get pains in my stomach because I haven’t got any work. Caregiver (father, age 46).

### Educational provision

Education was the key to upward social and economic mobility; as one 14-year-old girl stated “my father and his brother didn’t study, now they have low-paying jobs.” Consequently, children expressed anxieties about their class ranking, passing exams, and fulfilling the dream of securing a university degree. The competition to be “first in the class” [*awwal numra*] was enormous, and emblematic of the premium placed on scholastic success:

I have the fourth position in the class, but I want to be the *awwal numra*. I’m always thinking of ways to defeat the guy who is *awwal numra* so I can take his place. I don’t like his face, every time I look at him I ask God to kill him so I can take his place. Student (boy, age 13).

**Fig. 2:**
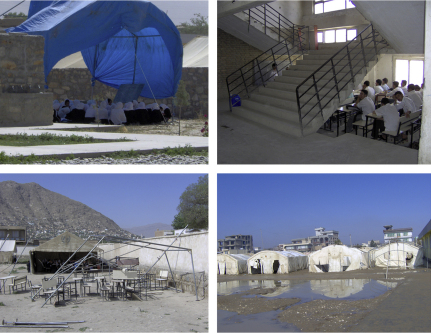
Examples of school classroom conditions: overcrowding (class squeezed into stairwell) and overspill in outdoor tents.

Children struggled to obtain an education despite difficult, often obstructive school and home environments; “I cannot learn” was a common expression of such frustration. Overcrowded schools lacked sufficient chairs, desks, heating and ventilation, drinking water, and toilets. Classes held in tents exposed students to cold, rain, heat, and dust (Fig. 2). Study at home was frustrated by a lack of heating/lighting, the non-literacy of caregivers, and parental demands for children to assume time-consuming chores, such as replenishing household water supplies, or carpet-weaving for 4–5 hours before/after a half-day at school. Students walked to school on dusty, muddy, or polluted roads, arriving dirty and exhausted; in cities, they faced heavy traffic, harassment by *badmash* [ruffians], verbal abuse from bus conductors, and the perceived threat of kidnappers [*adamdozd*].

Children also criticised the quality of educational provision. Course materials, books, and basic teaching supplies were absent or in short supply. Teachers were “unqualified,” due to the low salaries set by the government; as one 15-year-old boy explained “they’re so low that all the qualified teachers are working for NGOs.” The fact that teachers left school during class time to work at second jobs irritated students who walked for 1–3 hours to attend class.

### Governance and social justice

I’ll tell you one thing- I don’t have anything good to say about the Taliban or Najibullah or Masood or any of the *mujahideen*… We need peace. We need food. Whoever provides us with these… we need him. If someone asks me who I support, I will say ‘none of them.’ They all strive for power for themselves. Look at their relatives, look at the luxury cars they have, look at the big houses they’ve built! Powerful people get all the money, the poor stay poor. Caregiver (60-year-old male war veteran).

Issues of governance and social justice permeated the day-to-day lives of respondents. Political instability and corruption were linked to the inadequate provision of electricity, water, clinics, roads, and schools. Itinerant street-traders reported bribing traffic police in order to work; one 12-year-old boy described this as “torture,” and reported that “I’m always afraid when I’m working.” There were accounts of bribery in the judicial system, the payment of “kickbacks” to secure employment, and the need for wealth and influence to pass exams or obtain a place at university. As one 18-year-old male put it “Rich people get to go to university, poor people don’t have the right to.” Others reported being denied advancement because they lacked a *wasita* [personal connection] to “pull strings” in their favour; thus a 24-year-old male stated, “if you don’t have a *wasita* then talent counts for nothing.” The inability to negotiate this system produced disillusionment and resentment:

I passed the university entrance exam twice, but I never got any results from it, I wasn’t admitted to a faculty. Now I am discouraged and I don’t want to study. I don’t want to get a higher education any more. Hard work and effort are useless in our country. Caregiver (aunt, age 22).

## Overview of solutions

**Fig. 3:**
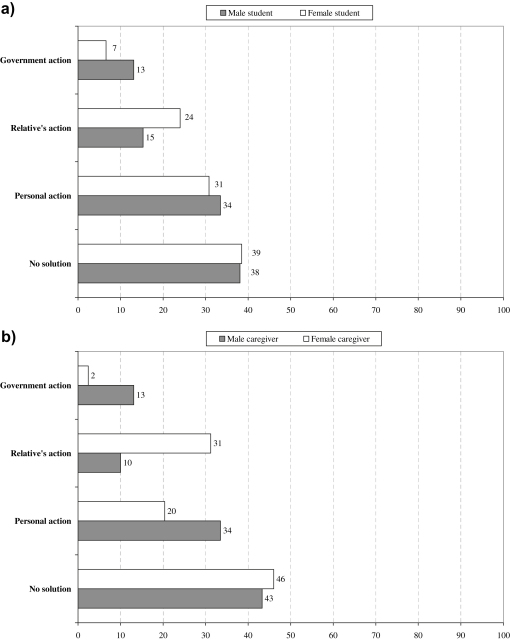
**a & b:** Types of solutions envisaged by 1011 students and 1011 caregivers (% of responses) (a) students, by gender (b) caregivers, by gender.

Some 40% of respondents expressed powerlessness in response to their situation, while others saw a solution rooted in personal, a relative’s, or government action (Table 1: Q4). The distribution of responses from students and caregivers was strikingly similar (Fig. 3), but there were substantial gender differences. Women more often identified intervention by a relative, while men looked to personal or government action.

Two key reasons – a dearth of education and social justice – underpinned the statements of those who felt powerless to overcome life stressors. For men, illiteracy was seen as an insurmountable constraint on better employment and income opportunities. As a 48-year-old driver stated: “I’m illiterate and I don’t want my children to be illiterate. An illiterate person can’t do anything.” For women, this was compounded by cultural constraints placed on mobility and behaviour: one 26-year-old, whose husband was unemployed, stated that she could do nothing to overcome their “broken economy” because “I am from a conservative family and they won’t allow me to work.”

Similarly, education and employment underpinned statements from those who saw a solution to their problems in personal or a relative’s action. For men, this was tied to securing steady work and “working hard” to make ends meet while their children obtained an education. A common demand was that the government should “provide jobs for the people” or “build factories for people to work in.” Women placed responsibility for improvement squarely upon the shoulders of their husband or an elder son, who were expected to “find a good job and have a good income.” Both boys and girls saw education as a solution for the entire family, stating that “education is the only way to improve my life.”

## Cultural values and social hope

The only way to make life better is to be hopeful. If a person has hope, then he or she can work and acquire knowledge to make their life better. Caregiver (mother, age 49).

**Fig. 4:**
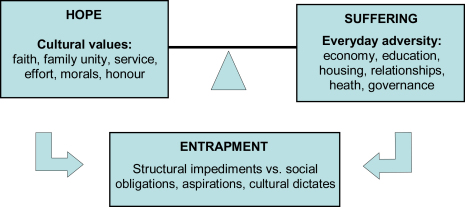
Cultural understandings of distress and resilience in Afghanistan: suffering, hope, and entrapment.

Thematic analyses revealed six key cultural values in responses to the open-ended question about how “life could be better” (Table 1: Q5). They underpin a discourse which counterbalances narratives of suffering and despair (Fig. 4).

### Faith [iman:  ]

Strong religious conviction- *iman* – was clearly a source of individual strength in the face of misfortune, and one which crossed generations. A 43-year-old tenant farmer, supporting a household of ten, expressed the centrality of this in verse:

Better a poor servant of God than a rich man without *iman*

A life without serving God is a shameful and uncertain one.

*Iman* was articulated in expressions of resignation – an acceptance that life was determined by God and, ultimately, beyond individual control; as one 63-year-old schoolteacher stated, “maybe it is our destiny to never own a home.” Adults often rounded off statements of powerlessness with the phrase “God is kind” [*khodawand mehraban ast*], elaborating that “only God knows when He will give me a comfortable life.” Expressions such as “it is up to God to decide” and “I can’t do anything except be patient with God and wait for Him to help” were typical across generations. Thus one 65-year-old father explained that “humans don’t have any option but to accept God’s will… there isn’t any way to improve our lives aside from having *iman,*” quickly followed by “I hope that God will solve my debt problems.” An 11-year-old girl felt that “if God wants our life to be better, it will get better… if not, it won’t.”

*Iman* was a central component of an imagined future, one which depended upon the beneficence of God, who would reward those who demonstrated *iman*, and punish those who did not. As a 14-year-old boy who worked after school in a grocery store put it: *awwal khoda, duom paisa* [first God, then money]. Similarly, a 22-year-old male stated: “It is up to God. We simply try our best and don’t do any bad things, like robbery or gambling or opium,” while *iman* was the foundation of hope for a 35-year-old woman, who stated: “Only God solves our problems… I want to have a strong *iman*. I want God to give me a son.”

### Family unity and harmony [wahdat:  and ittifaq:  ]

Family unity and harmony were articulated as the ability to achieve consensus in decision-making, peacefully resolve disputes, and share a household without conflict. The importance of these values across generations is clear in the following statements:

I’m living in peace with all my brothers. As long as there is *ittifaq* between us everything’s fine, but when there is no *ittifaq*, then nothing is good. Caregiver (grandfather, age 73).

My father is tormenting my mother and my sisters. There used to be twelve of us at home, but my brothers and sisters left because of my father… I kept praying to God to kill my father… I want God to change my father’s nature and give my family *wahdat*. Student (girl, age 16).

Successful economic activity depended upon maintaining co-operative family relationships across generations. Strong *wahdat* and *ittifaq* maximised employment opportunities and access to credit, effective decision-making regarding the allocation of wealth and property, and arranged marriages. Loss of family unity and harmony had profound consequences for material and psychosocial wellbeing:

A few months ago we had a fight with our uncle’s family. It was just a verbal quarrel, but it upset my family. The story is that we came back from Iran with my uncle’s family and we bought some land together. We built a house on it, but after we’d lived in the house for a year my uncle kicked us out. This really upset me. I can’t forget his mistreatment of us. Caregiver (sister, age 20).

### Service [khidmat:  ]

The value of ‘service’ lay at the core of future aspirations. Students wanted to complete an education and acquire a job in order to “serve” their parents, family, and community, as in “I want to get a higher education and serve my people” or “I want to be a doctor and serve the country.” The term was deployed by adults when voicing expectations for children:

I want my children to grow up, and my daughters to get married. I hope God keeps my children on the path of the righteous. I want them to have good health and serve their father and mother. Caregiver (mother, age 42).

Service to family meant fulfilling obligations to contribute to the household economy, obedience to parents, and a duty to eventually support them. As one 15-year-old boy plainly stated: “We expect our parents to provide us with the things we need, and they expect us to serve them.” Failure to “serve” was a source of shame, and a flaw in character; for example, a 40-year-old mother expressed disappointment with her two married sons because “they don’t help me, or serve me and my children.”

### Perseverance and effort [koshesh:  ]

Respondents referred to *koshesh* when asked to describe how they might overcome particular stressors – finding a steady job, or balancing the demands of work and school. Statements such as “work and *koshesh* can make our life better” or “education and *koshesh* will improve our lives” were typical from children, while adults echoed this with phrases such as “our life can only get better through hard work and *koshesh.*” *Koshesh* was also embedded in wider economic and social aspirations, cascading across generations:

I want Allah to cherish my 10-year-old son and give him a high-ranking position to solve our problems. My son should study and work hard to make our life better after his graduation from school… if my children get high-ranking positions this will change my life… I pray to Allah to solve our problems, to improve our economic position, to give us a house of our own. Caregiver (mother, age 45)

There were many such testimonies to the value of *koshesh* in fighting both war-related and economic adversity. For example, one 39-year-old farm labourer, who experienced shelling, house-burnings, and the execution of neighbours and relatives during the Taliban period, stated that “God is powerful – if there is more rain, and I can rent more land next year, I will work hard, and our problems will be solved.” Similarly, a 16-year-old boy, who wove carpets from 4am until noon, attended school during the afternoon, and returned to his loom afterwards, stated that the solution to his family’s “broken economy” was that he had to “work harder and not take as much time to rest.”

### Morals [akhlaq:  ]

Morals referred to all codes governing appropriate behaviour – deference to one’s parents and community elders, modesty in dress and comportment, and good manners in day-to-day relationships. Along with education, *akhlaq* was essential for a successful future. Statements from children such as “education, training, and having good morals can help you progress in the future” were typical in this area, and dovetailed with parent’s often-expressed desire that children would have “good morals and strong faith” in order to “uphold the honour and respect of their family.”

To have “good *akhlaq*” was also a marker of worthiness and character, while the loss of *akhlaq* was identified as a consequence of economic hardship and domestic conflict. As a 40-year-old mother stated: “We have a bad economic situation, and this has ruined our *akhlaq*. My husband is a good person, but when it comes to money, he gets emotional and fights.”

### Social prominence, respectability, and honour [‘izzat:  ]

Ultimately, adherence to cultural values was a path to respect and social recognition. Parents often wanted their children to be *mashour* – literally, “famous” or “prominent” – meaning that they wanted them to be upstanding, respectable members of their community, who exhibited *iman* and *akhlaq*, and worked hard to “serve the people.” Children expressed this ambition in statements such as “I want to fulfil my parents’ wishes for me to be *mashour* in the future,” or “I want to be a good Muslim and an important person in my community.” Social position was also key in the choice of marriage partners; as a female 20-year-old stated, “I can bring changes to my life by having good morals, good behaviour, and a good job. I want to marry someone with a good character who is also *mashour*.”

The desire for social respectability was closely tied to the deeply-rooted need to maintain personal and family honour. By fulfilling their parent’s ambitions for them, by working hard, demonstrating good morals, and serving others, children would maintain this. Children expressed anxiety about not doing well in school because they “would have to be ashamed” in front of their relatives. Similarly, a 40-year-old mother was distressed by her husband’s objections to a marriage she had arranged for her son, saying “if I don’t marry him with my niece, everyone will think I am a bad person.” This cultural imperative is also evident in a statement from a woman whose husband repeatedly abused her:

Last year my husband kicked me out of our house. I spent a month at my father’s home without my children. Now every day he’s telling me to go away, that he doesn’t need me around any more. But I suffer all these tortures because of my children and my father’s honour [*‘izzat*].

## Sources of conflict and forms of entrapment

While the desire to maintain key cultural values underpinned expressions of fortitude and hope, our data make clear that this also generated feelings of entrapment, friction within families, and personal distress. Three forms of entrapment were manifested in conflicts affecting everyday life and cross-generational interactions. One stemmed from individual or collective inability to demonstrate cultural values and meet social obligations, primarily as a consequence of economic hardship. Another existed where individual aspirations to achieve social and cultural milestones came into conflict with expectations inherent in the values of morality, service, and family unity. Finally, cultural dictates surrounding marriage decisions, the social position of women, and the dynamics of collective households were sources of suffering *per se*.

### Poverty and the loss of honour: the trap of social obligations

Adults often expressed their *material* poverty in terms of being unable to fulfil *social* obligation*s*, with consequent loss of honour. The inability to own a home, provide a stable income for the household, and arrange preferred marriages for children, were common sources of distress, shame, and depression. Poverty also undermined family unity; it prevented families from reinforcing their relationships with relatives through the exchange of food and gifts, or attendance at important family gatherings. As one 45-year-old father put it: “we haven’t got enough money to have a relationship with our relatives,” while another reported a complete rupture for similar reasons:

Our relatives verbally abused us, so we moved away from them five years ago. I was proposing to arrange a marriage between a girl of theirs and my brother, and they insulted us a lot. They said we were poor and had no business asking for one of their girls. So we moved away, it still upsets me when I remember what happened.

Asking for financial assistance from peers, compelling women and children to work outside the home, and begging or incurring debts, were also economically-induced sources of shame. One grandfather went as far as to say that it was “better to be hungry than to go into debt,” while a father stated he was “committing a sin” by forcing his children to weave carpets at home. A 28-year-old uncle summarised the situation as follows:

It’s a sort of custom or common practice of people, that when you are poor, they no longer treat you like a human being – they don’t respect you or care about you – you’re worthless because you haven’t got money.

The potential mental health consequences of such feelings of social inadequacy and marginalisation were reflected in statements such as “death is better for me,” expressed by the 38-year-old wife of a marketplace cart-pusher, or “I’m only staying alive for the sake of my children, otherwise I’d rather be dead,” from a 45-year-old schoolteacher. In a culture which places a premium on large, extended families, interlinked by marriage and the exchange of wealth, a “broken economy” was clearly debilitating for both social functioning and psychological wellbeing.

### Curtailment of education: the trap of social aspirations

**Fig. 5:**
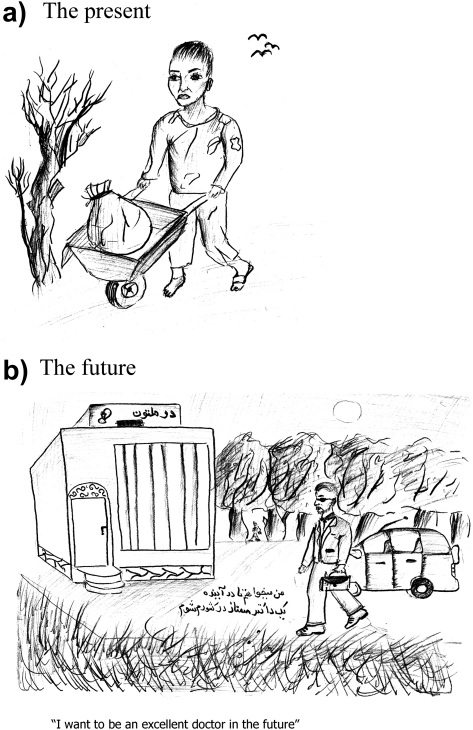
Drawings by a 14-year old boy taking art classes at school. (a) His life in 2006, characterized by economic difficulties (he works odd jobs to earn money for his household). (b) His life in the future (he carries a medical bag from his car to the workplace).

Students worried about curtailment of their education due to poverty or cultural directives. Both boys and girls placed an extremely high premium on attaining academic qualifications, seen as leading to salaried positions as teachers, doctors, or engineers. The significant gap between present circumstances and future aspirations was evident in students’ drawings, exemplified in Fig. 5.

However, such personal ambitions were often squeezed out by pressing economic needs and the cultural demand to maintain family unity:

I’m the eldest son in my family. I had to quit school because of life’s problems. We’ve got six people in our home, and I am the only one working. Whenever I see other boys my age going off to school I get upset, because I had to leave it behind to support my family. Caregiver (brother, age 19).

For some girls, life’s most important problem was the “pressure” placed upon them by a father, uncle, brother, or mother to stop going to school, or to avoid the pursuit of subjects that conflicted with parental expectations. They were told that school was “a waste of time,” or not to attend classes in “English and computers” because they were “full of boys.” A typical statement in this regard comes from a 65-year-old father: “We don’t have the practice of letting our women go to school. When we marry them off, their owners will feed them.” The cross-generational force and gender dimension of this conflict is clear from the following statement:

I’ve been having arguments about my sister, and I am worried for her. My eldest brother is 18 years old, he’s studying in the 12th grade. He’s trying to stop my sister going to school. When I was in 4th grade, my paternal grandmother and my unmarried aunt told my dad to stop me from going to school. Now they’re doing the same thing, telling my brother to stop my sister. I’m really worried about her. I wanted to have an education, now I have this wish for her. Caregiver (sister, age 18).

In addition to economic impediments and cultural dictates, the threat of political violence and the “return of the Taliban” added to anxieties regarding unfulfilled educational ambitions:

I worry about my future. During the wars my education was interrupted and now I am behind in school. Kabul has security problems these days, my father says the situation is very bad here. I’m afraid that if it gets any worse, he won’t give us permission to go to school any more. Even if the security situation improves, I’m worried that my father won’t let me continue past the 12th grade, and I want to go to university. Student (girl, age 16).

Frustrated aspirations led to conflict, distress, and despair that could be deeply injurious to health:

Because we have economic problems, my father forced me to quit school. So I swallowed rat poison after that, and I was in hospital for a week. They pumped my stomach out and I couldn’t eat for nine days. Caregiver (brother, age 18).

### Marriage, collective households, and structural violence: the trap of cultural dictates

Marriage arrangements were often a source of conflict within and between families – and a form of entrapment. For children, the value placed on exhibiting good moral character and service to one’s parents was often at odds with personal preferences regarding choice of spouse and timing of wedding. Failed attempts to arrange a marriage led to considerable distress, as was the case for a 31-year-old man, who recollected that “nine years ago I wanted to kill myself, because I wanted to marry my maternal cousin, but they married her off to another man,” or a 43-year-old mother who reported heated arguments with her son over his desire to marry a girl he loved, and stated, “it’s been a year that this has been going on and I’ve had *takleef asabi* because of it.” There were also expressions of frustration with cultural prescriptions in this area, particularly from young women; as one 16-year-old girl bluntly stated: “I wish parents would let their children go to school and not just marry them off when they’re young.”

The culturally-prescribed practice of living in a collective household with a husband’s relatives was also a source of suffering for married women. Many reported experiencing verbal and/or physical abuse, from male and female in-laws, due to their failure to ‘serve’ their husband’s family. The tacit acceptability of such ‘disciplinary’ violence is exemplified in the following statement from a 15-year-old boy:

My uncle beats my mother. This is sometimes what happens among people, if the father is away from home, the uncle beats younger members of the family or the women. This is normal, for an uncle or cousin to beat his brother’s wife. In Afghan villages, it’s normal for a husband to beat his wife.

An example of the severe consequences such violence has on women comes from a 36-year old mother, placed into an arranged marriage from childhood:

The reason I tried to kill myself was that my husband and my mother-in-law and father-in-law were constantly beating me, severely beating me. I was thirteen years old when I was married, and in the first years they beat me a lot because I hadn’t had a child, that’s when I decided to throw myself off the roof, because of all the beatings. Then, when I did have children, they would beat me because they were all girls, because I hadn’t produced a boy. That’s when I decided to take all the medicine. In the end I had a boy, and they stopped beating me.

Within collective households, other generators of distress and potential violence included tensions within polygamous families related to the distribution of wealth between co-wives and their children, failed ‘exchange marriages’ – in which two families agree to swap women as brides for their sons – and protracted disputes between relatives over the rights to shared property and inheritance.

A perceptive critique was voiced regarding the links between cultural ambitions, achievable with economic success, and the perpetuation of social injustice for women:

Life can get better by having good morals, patience and education… and having money… but unfortunately having a lot of money is bringing misery to my mother, because my father will use it to marry a second wife. Caregiver (sister, age 19).

## Discussion

Our data speak to three dimensions of everyday life in Afghanistan. They present poignant testimonies of everyday adversity and cross-generational suffering. They demonstrate that hope and fortitude is founded on the expression of fundamental cultural values that give order and promise to life. They reveal sources of entrapment, as families struggle to adhere to their values in the face of pronounced structural inequalities injurious to well-being. These three dimensions address key issues in the current literature on social suffering and resilience in conflict-affected areas, as discussed below.

In contemporary Afghanistan, material poverty lies at the root of *social suffering*, and drives a multi-faceted discourse around it. The “broken economy” [*iqtisad kharab*] is the root of *all* miseries, a struggle that goes well beyond the lack of food, clothing, and adequate shelter. Economic insecurity produces complex tensions within families: it is a central driver of psychological distress, physical pain, domestic violence, and community conflicts. It is also a key impediment to the achievement of social and cultural ambitions. Afghans articulate mental and physical ill-health as both a cause and a result of material poverty, as poverty morphs into multiple experiences of suffering, both individual and collective. This exemplifies what Kleinman called *the violences of everyday life* – present in multiple and insidious forms, at the level of personal experiences, cultural norms, and routine social coercion (Kleinman, 2000: 238). Drawing from both child and adult narratives, our data exemplify how local understandings of well-being are more closely tied to everyday experiences of structural violence than to past experiences of war, and how everyday suffering cascades from one generation to the next, the result of close interdependence between family members and shared experiences of adversity.

Resilience and fortitude rest upon a sense of hope: the belief that adversity can ultimately be overcome and a process of ‘meaning-making’ that gives coherence to past, present, and future experiences. In Afghanistan, the bedrock of this hope is the production and maintenance of a set of long-lived cultural values. Strong religious faith [*iman*] and individual effort [*koshesh*] are values that structure a *discourse of resilience* in the face of adversity, often through acceptance of “the will of God” and a hope that everyday perseverance will be rewarded with His “mercy” and “protection.” Strong relationships between hardship, faith, and endurance, tinged with fatalism regarding matters of health and socio-economic success, were also highlighted by Canfield (1975: 483) among famine-stricken Hazara communities in the 1970s. While hope, fatalism, and perseverance are complex facets of religious discourse, our data indicate that professions of powerlessness do not equate to a sense of hopelessness and a loss of faith. Thus 40% of our respondents saw ‘no solution’ to key life stressors, but went on to express statements of religious conviction and fortitude. Three other constructs – service, morality, and family unity [*khidmat; akhlaq; wahdat*] – complement *iman* and *koshesh* to give order and meaning to personal experience and social relationships: these values underpin honour [*‘izzat*] and respectability, key to social functioning and individual dignity, and core *psychosocial* dimensions of resilience.

By and large, Afghan families suffer great material poverty, but not a poverty of aspirations: on the contrary, hard work and education are seen as the gateway to economic security and social respectability. Our data make clear that Afghan children internalise the importance of scholastic success, of service to elders, and conformity to cultural codes governing morality. This resonates with the emphasis placed on *tarbia* [lit: training, upbringing] in a seminal qualitative study of Afghan families in Kabul (De Berry et al., 2003). As in other war-affected settings, a large component of resilience against the ‘hurt’ of social disadvantage comes from a determination to adhere to cultural values and life goals focused on seeing children well-educated and well-married (Loizos, 2008). Among Afghans, *social hope* cascades across generations, spurred on by promises of international assistance, reconstruction, and education.

Cultural values, however, are sources of *entrapment* as well as resilience, given the structural impediments to their realisation: poverty, ineffective governance, social injustice, and ongoing militarized conflict. At an individual and family level, strain arises due to an incompatibility between the desire to maintain personal or collective values and the bald economic demands of survival. Structural constraints place huge impediments on children’s ability to succeed in school and study at home, leading to frustration and disappointment. For many young people, ‘service’ to family will involve interrupting further education and sacrificing personal ambition, as poverty and cultural dictates compel adolescent sons to work and daughters to be married. The inability to fulfil one’s social obligations and to realise personal aspirations are twin facets of entrapment – broken dreams generated by the broken economy. Paradoxically, the ability to demonstrate adherence to cultural values may reproduce inherent social injustices, perpetuated for women by the dominance of men in politics, economics, and social relations, and for youth by the power of elders in decision-making.

In this sense, culture is not just an anchor of resilience, but also an anvil of pain. On one hand, the profession and maintenance of cultural values is central to the construction of social identity, order, and hope. On the other, inability to conform to cultural dictates is source of great psychosocial distress. Failure or frustration in attaining social and cultural milestones lies at the root of social suffering and mental ill-health, as articulated in local idioms of stress, anxiety, and depression, or conflicts that are debilitating and life-threatening. Culture becomes a double-edge sword, as argued for religion by Wessells and Strang (2006: 200), in being both a source of violence as well as a resource for social functioning. Our data show the frustration and loss of self-worth experienced by Afghan men unable to provide adequate support for their families. Women and children report this as a serious driver of strain and violence in the family. Similar observations linking shame, frustration, and violence were made by Mollica (2006: 173) when describing Cambodian refugee experiences: jobless fathers were “humiliated in the eyes of their children, their neighbours, and themselves,” felt “castrated and impotent,” while wives “become enraged at a husband who cannot support the family.”

Some sense of the strength and durability of Afghan cultural values, and their role in the production of inequity and social tensions, can be found in Tapper’s work (Tapper, 1991) in a northern Afghan community in the 1970s. Highlighting the central role played by honour in all decision-making regarding resource allocation – labour, land, women, debt, and credit relations – Tapper described “the real inequality in such a system as a spiral whereby the weak lose control of resources of *all* kinds, lose honour, and become weaker still, while the strong gain control of resources, gain honour, and become stronger (p. 299).” She linked women’s jinn-possession to a form of “social rebellion” (p. 217), but revealed that those in socially weak positions would be dismissed as faking insanity or possession. Similarly, Dupree (1980) observed that in Afghanistan women “sometimes crack under the strain of combined boredom, frustration, and mistreatment” (p.125) and that mental health problems among men and women arose from frustrated cultural expectations (p. 190). In our study, Afghan narratives embody both the dramatic violence of war, specifically, grief [*jighar khun*] related to war trauma, and the structural violence of poverty and marginalisation, such as irritability, agitation/lethargy, and anxiety [*takleef asabi, fishar payin/bala, tashwish*] arising from everyday economic and social stressors.

Cultural affiliation, ideological commitment, and networks of social support are usually understood as protective to youth and adults in conflict zones (Seginer, 2008; Wexler et al., 2009). In particular, culture and religion provide strength and solace in environments where military and civil institutions fail to provide social justice (Aggarwal, 2007). Pursuing this line of argument, mental health intervention programmes in conflict zones are currently urged to build upon community contexts and cultural norms to enhance the relevance and effectiveness of psychosocial support (Betancourt & Khan, 2008: 323; IASC, 2007). In Afghanistan, community psychosocial support interventions are burgeoning (De Berry, 2004) on the back of large-scale investment in public health and education. However, given that crises are chronic and governance has little “clarity of purpose,” the family has proven to be the only stable institution available (Dupree, 2004). Certainly, culture ‘matters’ to resilience in war-affected areas: our data demonstrate both how cultural values work – creating meaning and imparting moral and social order – and why they work – generating hope to overcome social suffering and everyday violence. Yet in the absence of a functioning economy and equitable access to basic resources, efforts to promote cultural values can entrap those in a position of vulnerability and powerlessness, while efforts to promote child education arguably raise hope and expectations to the point of illusion and assured frustration.

Our study gives qualitative depth to epidemiological studies demonstrating the impact of daily stressors on stress and mental health in conflict zones (Miller et al., 2008; Miller & Rasmussen, 2010; Panter-Brick et al., 2008) and the associations found between child-caregiver wellbeing (Panter-Brick et al., 2009). In its focus on hope, it also addresses the complex issue of how to promote social cohesion in a society devastated by suffering and loss (Pedersen, Tremblay, Errázuriz, & Gamarra, 2008: 214). With specific reference to resilience, our findings resonate with work arguing for the need to identify, through detailed ethnographic work, what social policies will improve living conditions, what counts as success in the eyes of marginalised groups (Canvin, Martilla, Burstrom, & Whitehead, 2009), and “what really matters” in living a dignified and moral life amidst uncertainty and danger (Kleinman, 2009). In social policy and public health, arguments that a ‘spiral of disadvantage’ engulfs poor families and that social inequality is injurious to wellbeing are now well-established (WHO, 2008). In conflict zones, these issues need fleshing out with both qualitative and quantitative work, to understand how resilience is specifically constructed in contexts of violence, economic deprivation, and social oppression (Panter-Brick, 2010). Our data indicate that interventions focusing on everyday social ecology – strengthening family and wider social networks – need to go hand in hand with interventions focusing on everyday material ecology – altering daily economic stressors that are the nexus of social suffering. They also indicate that the quality of education provision must be improved to further goals of social justice and psychosocial wellbeing in the lives of a new generation.

This touches on the *ethics of hope*, a critique of post-Taliban reconstruction efforts in Afghanistan akin to Hage’s trenchant critique of global capitalism. In a context of widening social inequalities, maldistribution of capital, and inequitable state policies, people suffer from a shrinking configuration of hope, a sense of entrapment in ‘going nowhere’ in terms of existential and social mobility (Hage, 2003: 12–21). The programme of massive refugee repatriation (Turton & Marsden, 2002) and the ‘Back to School’ campaign in Afghanistan (Hunte, 2006) are two examples of hope-building policies that have raised expectations without sufficient follow-through to create lasting socio-economic opportunities and mitigate deep-set inequalities. In this study, Afghans articulated a forceful, policy-relevant message: there is no health without mental health, no mental health without family unity, no family unity without work, dignity, and a functioning economy, and no functioning economy without good governance. More ethical and realistic policy goals (UNDP, 2004: 196) would need to address the everyday priorities of ordinary Afghans, who underscore the structural violence of poverty rather than the dramatic violence of war, and the importance of maintaining personal and social dignity as the key to a hopeful future.

## Figures and Tables

**Table 1:** Protocol and analysis of a ‘Problems and Solutions’ semi-structured questionnaire, implemented with 1011 students and 1011 caregivers in Afghanistan.

Questions to elicit ‘top-of-mind’ concerns, main problems faced in day-to-day life, and solutions envisaged	Thematic analyses and categorisation of respondent statements
1. Now I would like you to talk to me about your day-to-day life here. In particular, can you tell me the kinds of problems you face – the things that make you worry, or make you nervous or upset, or just irritate you… Can you tell me, what are your main problems or worries these days? (record three) 2. Which problem or difficulty bothers you the most? (record response) 3. How much does this problem affect your day-to-day life? (not at all, a little, a moderate amount, a great deal). 4. What can you do to overcome this problem? Is there any way to solve it? (record response) 5. Tell me… what are some ways that your life could be better? If you could change anything in your life right now, what would it be? (record response) 6. Thank you for taking the time to answer these questions. Is there anything else you want to add? (record response)	(1–6) Review of all verbatim statements, written down by fieldworkers in Dari/Pashto, by the project manager after interviews each day; translation into English by professional translator in Afghanistan; final review by the project manager before dispatching data; manual processing by first author in vernacular and English language, for computerised data entry; review of all data in English format by both authors. (3) Impact on daily life (data not shown) (1–2, 4–6) Thematic analysis of translated statements; marking up keystatements and emblematic responses regarding adversity, suffering, and fortitude. Contextualisation in light of reported health and traumatic life events. (1–2, 4–6) Responses categorised into domains (by authors, after thematic review) for quantitative illustration. Problems categorised as relating to: the economy, housing, health, school, social relationships, and governance. Solutions categorised as: none envisaged, action by self, action by relatives, or action by the government.

Child and adult respondents were interviewed, separately and in a private location, in their own language by the same interviewer.

## References

[bib1] Aggarwal N.K. (2007). Exploring identity, culture, and suffering with a Kashmiri Sikh refugee. Social Science & Medicine.

[bib2] Almedom A.M., Glandon D. (2007). Resilience is not the absence of PTSD any more than health is the absence of disease. Journal of Loss & Trauma.

[bib3] Almedom A.M., Summerfield D. (2004). Mental well-being in settings of complex emergencies: an overview. Journal of Biosocial Science.

[bib4] Bakhshi P., Trani J. (2006). Towards inclusion and equality in education? From assumptions to facts.

[bib5] Barber B.K. (2008). Contrasting portraits of war: youths’ varied experiences with political violence in Bosnia and Palestine. International Journal of Behavioral Development.

[bib6] Barber B.K. (2009). Adolescents and war: How youth deal with political violence.

[bib7] Bernard H.R. (2006). Research methods in anthropology; qualitative and quantitative approaches.

[bib8] Betancourt T., Khan K. (2008). The mental health of children affected by armed conflict: protective processes and pathways to resilience. International Review of Psychiatry.

[bib9] Boyden J., de Berry J. (2004). Children and youth on the front line: Ethnography, armed conflict and displacement.

[bib10] Canfield R.L., Williams T. (1975). Suffering as a religious imperative in Afghanistan. Psychological anthropology.

[bib11] Canvin K., Martilla A., Burstrom B., Whitehead M. (2009). Tales of the unexpected? Hidden resilience in poor households in Britain. Social Science & Medicine.

[bib12] Carbonella A. (2003). Towards an anthropology of hope. Focaal.

[bib13] Cardozo B.L., Bilukha O.O., Crawford C.A.G., Shaikh I., Wolfe M.I., Gerber M.L. (2004). Mental health, social functioning, and disability in postwar Afghanistan. Journal of the American Medical Association.

[bib14] Davis J. (1992). The anthropology of suffering. Journal of Refugee Studies.

[bib15] De Berry J. (2004). Community psychosocial support in Afghanistan. Intervention.

[bib16] De Berry J., Fazili A., Farhad S., Nasiry F., Hashemi S., Hakimi M. (2003). The children of Kabul: Discussions with Afghan families.

[bib17] Donini A. (2007). Local perceptions of assistance to Afghanistan. International Peacekeeping.

[bib18] Dupree L. (1980). Afghanistan.

[bib19] Dupree N.H. (2004). The family during crisis in Afghanistan. Journal of Comparative Family Studies.

[bib20] Farmer P. (2004). An anthropology of structural violence. Current Anthropology.

[bib22] Hage G. (2003). Against paranoid nationalism: Searching for hope in a shrinking society.

[bib23] Havel V. (1990). Disturbing the peace.

[bib24] Hobfoll S., Watson P., Bell C.C., Byrant R.A., Brymer M.J., Friedman M.J. (2007). Five essential elements of immediate and mid-term mass trauma intervention: empirical evidence. Psychiatry.

[bib25] Hunte P. (2006). Looking beyond the school walls: Household decision-making and school enrolment in Afghanistan.

[bib26] IASC (2007). Guidelines on mental health and psychosocial support in emergency settings.

[bib27] Kleinman A., Das V., Kleinman A., Ramphele M., Reynolds P. (2000). The violences of everyday life: the multiple forms and dynamics of social violence. Violence and subjectivity.

[bib28] Kleinman A. (2009). What really matters: Living a moral life amidst uncertainty and danger.

[bib29] Kleinman A., Das V., Lock M. (1997). Social suffering.

[bib30] Loizos P. (2008). Iron in the soul: Displacement, livelihood and health in Cyprus.

[bib32] Mastern A.S., Flynn R.J., Dudding P.M., Barber J.G. (2006). Promoting resilience in development: a general framework for systems of care. Promoting resilience in child welfare.

[bib33] Miller K.E., Omidian P., Rasmussen A., Yaqubi A., Daudzi H. (2008). Daily stressors, war experiences, and mental health in Afghanistan. Transcultural Psychiatry.

[bib34] Miller K.E., Rasmussen A. (2010). War exposure, daily stressors, and mental health in conflict and post-conflict settings: bridging the divide between trauma-focused and psychosocial frameworks. Social Science & Medicine.

[bib35] Miyazaki H. (2004). The method of hope: anthropology, philosophy, and Fijan knowledge.

[bib36] Mollica R.F. (2006). Healing invisible wounds: Paths to hope and recovery in a violent world.

[bib37] Panter-Brick C. (2010). Conflict, violence, and health: setting a new interdisciplinary agenda. Social Science & Medicine.

[bib38] Panter-Brick C., Eggerman M., Gonzalez V., Safdar S. (2009). Violence, suffering, and mental health in Afghanistan: a school-based survey. The Lancet.

[bib39] Panter-Brick C., Eggerman M., Mojadidi A., McDade T. (2008). Social stressors, mental health, and physiological stress in an urban elite of young Afghans in Kabul. American Journal of Human Biology.

[bib40] Pedersen D. (2002). Political violence, ethnic conflict, and contemporary wars: broad implications for health and social well-being. Social Science & Medicine.

[bib41] Pedersen D., Tremblay J., Errázuriz C., Gamarra J. (2008). The sequelae of political violence: assessing trauma, suffering and dislocation in the Peruvian highlands. Social Science & Medicine.

[bib42] Pope C., Ziebland S., Mays N. (2000). Analysing qualitative data. British Medical Journal.

[bib43] Punamaki R.-L. (1996). Can ideological commitment protect children’s psychosocial well-being in situations of political violence?. Child Development.

[bib45] Rubin B.R. (2006). Afghanistan’s uncertain transition from turmoil to normalcy.

[bib46] Scheper-Hughes N., Bourgois P. (2003). Violence in war and peace.

[bib47] Scholte W.F., Olff M., Ventevogel P., de Vries G.J., Jansveld E., Cardozo B.L. (2004). Mental health symptoms following war and repression in eastern Afghanistan. Journal of the American Medical Association.

[bib48] Seginer R. (2008). Future orientation in times of threat and challenge: how resilient adolescents construct their future. International Journal of Behavioral Development.

[bib49] Stein D.J., Seedat S., Iversen A., Wessely S. (2007). Post-traumatic stress disorder: medicine and politics. The Lancet.

[bib50] Tapper N. (1991). Bartered Brides: Politics, gender and marriage in an Afghan tribal society.

[bib51] Turton D., Marsden P. (2002). Taking refugees for a ride? The politics of refugee return to Afghanistan.

[bib52] UNDP (2004). Afghanistan National human development report 2004: Security with a human face.

[bib53] Wessells M., Strang A., Boothby N., Strang A., Wessells M. (2006). Religion as resource and risk: the double-edged sword for children in situation of armed conflict. A world turned upside down: Social ecological approaches to children in war zones.

[bib54] Wexler L.M., DiFluvio G., Burke T.K. (2009). Resilience and marginalized youth: making a case for personal and collective meaning-making as part of resilience research in public health. Social Science & Medicine.

[bib55] World Health Organization (2008). Social determinants of health in countries in conflict; a perspective from the Eastern Mediterranean region.

